# Down-regulation of c-Myc following MEK/ERK inhibition halts the expression of malignant phenotype in rhabdomyosarcoma and in non muscle-derived human tumors

**DOI:** 10.1186/1476-4598-5-31

**Published:** 2006-08-09

**Authors:** Francesco Marampon, Carmela Ciccarelli, Bianca M Zani

**Affiliations:** 1 Department of Experimental Medicine, University of L'Aquila, L'Aquila, Italy

## Abstract

**Background:**

Expression of c-myc proto-oncogene is inappropriate in a wide range of human tumors, and is a downstream target of Ras/Raf/ERK pathway, which promotes c-Myc stability by enhancing c-Myc expression and activity.

The aim of this study was to investigate whether the oncogenic phenotype in the human muscle-derived Rhabdomyosarcoma (RD) cell line and in non muscle-derived human tumor cell lines (SW403, IGR39 and PC3) can be blocked by disrupting the c-Myc pathway either by means of pharmacological MEK/ERK inhibition or by direct inactivation of the c-Myc protein.

**Results:**

We demonstrate that, in all the tumor cell lines used, the MEK/ERK inhibitor U0126 rapidly induces c-Myc de-phosphorylation, which is followed by a marked reduction in its expression level, by inhibition of proliferation and by reversion of anchorage-independent growth. These data suggest that the targeting of pathways controlling c-Myc expression or stability reverses deregulated growth of different tumor-derived cell lines. Indeed, in RD cells, we found a marked down-regulation of cyclins E2, A and B and CDK2, all of which are known to be targets of c-Myc. Moreover, ectopic MadMyc chimera, a c-Myc function antagonist, causes dramatic growth arrest, CDK and cyclin modulation as well as inhibition of anchorage-independent growth in RD cells, as occurs in U0126-treated cells. In particular, we found that the mere inhibition of c-Myc by MadMyc chimera rescues the myogenic program, MHC expression and the acquisition of the myogenic-like phenotype in RD cells.

**Conclusion:**

Our data provide evidence of the key role played by the MEK/ERK pathway in the growth arrest and transformation phenotype of Rhabdomyosarcoma and of non muscle-derived tumor cell lines. In fact, MEK/ERK inhibitor, U0126, induces growth arrest, anchorage-dependent growth of these cell lines. In addition, the results of this study demonstrate that the direct inactivation of c-Myc by Mad/Myc chimera rescues myogenic program and leads to the reversal of the Rhabdomyosarcoma phenotype. In conclusion these data strongly suggest that the targeting of c-Myc by means of the MEK inhibitor can be tested as a promising strategy in anti-cancer therapy.

## Background

The Myc protein, which has been shown to play an essential role in the control of cell proliferation, growth, differentiation and apoptosis [1,2], is a member of the basic region/helix-loop-helix/leucine zipper (b/HLH/Zip) family of transcriptional regulators that is capable of both transactivation and transrepression [1,3] of a large number of target genes [4,5] through heterodimerization with its biological partner Max [6]. Members of the Myc family are activated in many, if not most, human tumors [1] and the strong selection for c-Myc over-expression in tumors appears to reflect the ability of c-Myc to provide constitutive signals that promote cellular transformation [2]. It has recently been reported that Ras controls c-Myc protein accumulation resulting from ERK-mediated stabilization of c-Myc by Ser62 phosphorylation, whereas subsequent Thr58 phosphorylation by glycogen-synthase kinase-3 (GSK-3) is required for c-Myc degradation [7]. Thus, Ras activates AKT, which in turn inactivates GSK3, leading to the block of c-Myc degradation pathway. Consequently, the frequent Ras mutations in human cancer [8] and concomitant deregulation of c-Myc suggest a possible synergistic relationship of c-Myc and Ras in the disruption of normal cell growth regulation [7]. Indeed, inhibition of the MEK/ERK pathway in v-Ki-ras rat fibroblasts, MDA-MB231 and HBC4 breast cancer cell lines, and c-Myc depletion by siRNA in MCF7 and over-expression of a c-Myc antagonist, Mxi1, in prostate carcinoma DU145, all induce reversion of the malignant phenotype [9-12].

Both the c-Myc and Ras/MEK/ERK pathways play an important role in the progression of the G1-cell cycle phase by enhancing cyclins expression [13,14] and CDK/cyclin complex activities [15,16]. In addition, c-Myc constitutive expression suppresses expression of the cell cycle inhibitors p21^WAF1 ^and p27^KIP1 ^[17].

Lastly, both c-Myc and ERK, as a consequence of their marked capacity to promote proliferation, play an important role in controlling the differentiation program in several cell type [1,2].

Interestingly, osteogenic sarcoma, harbouring conditional alleles of c-Myc, differentiate into mature bone under brief c-Myc inactivation [18]; likewise, transgenic mice that conditionally express c-Myc in liver develop hepatocarcinoma that is reversed following c-Myc inactivation [19]. Accordingly, the down-regulation of c-Myc results in the attenuation of both cell division and cell growth as well as in the protection against some apoptotic processes [20,21].

Given the synergistic relationship between MEK/ERK and c-Myc in cell growth and malignant transformation, the blocking of the MEK/ERK pathway [22] might conceivably be used against cancer.

The embryonal rhabdomyosarcoma cell line (RD) consists of muscle-derived precursors that fail to complete the differentiation program [23], probably owing to the action of mutated N-Ras proto-oncogene [24], mutated tumor suppressor p53 [25] and over-expressed c- or N-Myc [26].

Since we found that U0126, a MEK/ERK pathway inhibitor, induces p21^WAF1 ^expression [27] and promotes G1 cell cycle arrest and myogenic differentiation in RD cells [28], we decided to investigate whether the MEK/ERK pathway and c-Myc might cooperate in cell growth and transformation control in RD cells. Furthermore, in order to investigate the effect of MEK/ERK inhibition on non-muscle-derived cell lines we used colon adenocarcinoma- (SW403), melanoma- (IGR39), prostate-derived cell lines (PC3), all bearing mutated Ras and deregulated c-Myc [29-31].

We found that the disruption of the MEK/ERK pathway, by means of the MEK inhibitor U0126, dramatically decreased c-Myc expression level, inducing growth inhibition and reversion of anchorage-independent growth in all the cell lines used. Moreover, we show that direct inactivation of c-Myc by the MadMyc chimera protein, a repressor of c-Myc activity, causes growth arrest, reversion of anchorage-independent growth and myogenic differentiation in RD cells.

## Results

### MEK/ERK inhibitor drastically reduces c-Myc expression

**Figure 1:**
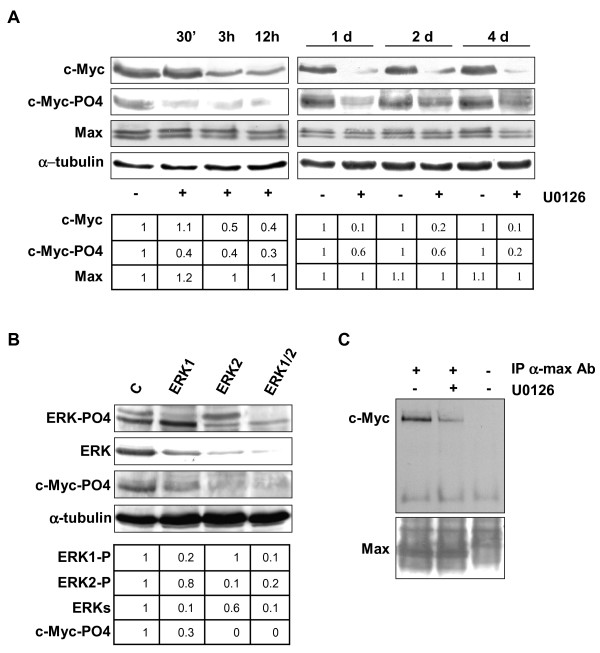
MEK/ERK inhibition affects c-Myc phosphorylation and expression in RD cells. **A**. Cell lysates from RD cells untreated (-) or treated (+) with 10 μM U0126 for indicated times were analysed by immunoblotting with specific antibodies for indicated proteins. α-tubulin expression shows the loading of samples. **B**. Cells were transfected with control (C) or ERK1, ERK2, ERK1/2 siRNAs and cultured for 3 days. Immunoblot of total lysates were performed using specific antibodies recognizing the indicated proteins. The values of fold increases over the control, arbitrarly set at 1, were obtained by densitometric analysis (A and B lower panels). **C**. Myc-Max heterodimer in RD cells untreated (-) or treated (+) with U0126 for 12 hours. Myc-Max complex was immunoprecipitated (IP) with a Max monoclonal antibody from extracts containing equal amounts of total proteins and subsequently analysed by immunoblotting with a c-Myc polyclonal antibody. Same filter was probed with a Max polyclonal antibody. Similar results were obtained in two different experiments.

In order to determine whether c-Myc is a target of the MEK/ERK inhibitor U0126 in RD cells, we performed time course experiments with 10 μM U0126 followed by immunoblotting. As shown in Figure 1A, U0126 induced early (3 hours), drastic (1d-4d) c-Myc down-regulation that persisted throughout treatment (up to 4 days). Owing to ERK inhibition (see additional file 1) [28], the level of phosphorylated c-Myc was markedly reduced (Fig 1A) before (30 min) c-Myc down-regulation began. That ERKs are upstream kinases of c-myc in RD cells, as suggested by U0126 experiments, was further demonstrated by RNA interference experiment with ERK1-, ERK2-, ERK1/ERK2-siRNA in transient transfection. After 3 days of transfection, we observed a down-regulation of total and phospho-ERKs and a lack of c-Myc phosphorylation particularly in ERK2 and ERK1/ERK2 siRNA transfected cells (Fig 1B).

While the expression level of Max isoforms (21 and 22 Kda), which heterodimerize with c-Myc [1], was unaffected (Fig 1A), the amount of c-Myc associated with Max was dramatically reduced in U0126-treated cells, as shown by immunoprecipitation experiments (Fig 1C). Equal amounts of Max were detected in the immunocomplex (Fig 1C). Taken together, these results indicate that c-Myc is a down stream target of ERKs and MEK/ERK inhibition mediates loss of c-Myc and of the c-Myc/Max heterodimer, providing one possible molecular mechanism of growth arrest i.e. that induced by the MEK inhibitor U0126.

### Effects of U0126 on G0/G1 arrest and cell cycle regulator expression in RD cell lines

**Figure 2:**
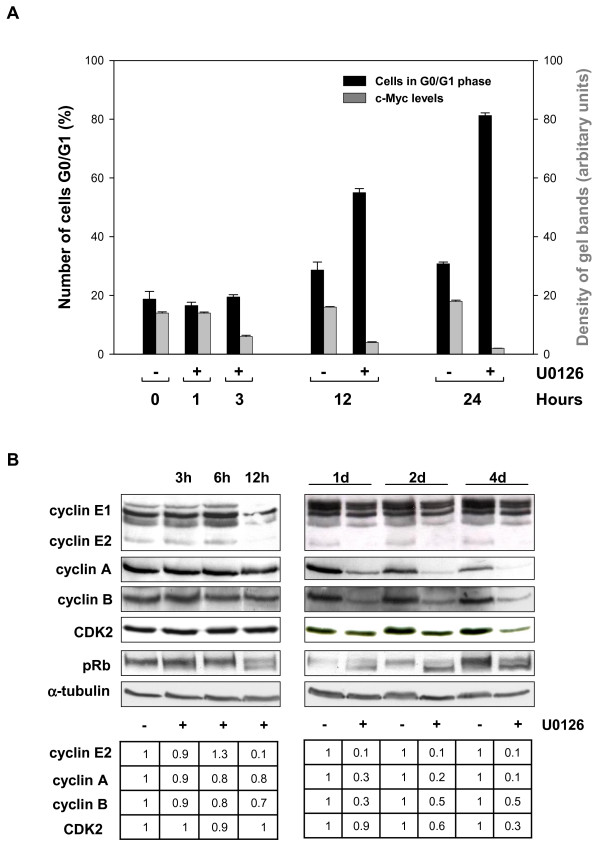
c-Myc down-regulation precedes the U0126-mediated effects on G0/G1 arrest and cell cycle proteins expression. **A**. Histogram showing the number of RD cells in G0/G1 phase expressed as percentage (black bars) and c-Myc levels (grey bars) expressed as arbitrary units (density of c-Myc band/α-tubulin band) in untreated (-) and U0126-treated RD cells (+) for the indicated times. Similar results were obtained in two separate experiments **B**. Cell lysates from RD cells untreated (-) or treated (+) with U0126 for indicated times were analysed by immuoblotting with specific antibodies for indicated proteins. α-tubulin expression shows the loading of samples. The values of fold increases over the controls, arbitrarly set at 1, are obtained by densitometric analysis (lower panel). Similar results were obtained in three different experiments.

Since c-Myc expression is well known to be down-regulated during inhibition of cell growth [32,33] we addressed whether the observed c-Myc down-regulation is simply a consequence of cessation of cell growth due to U0126 treatment. To this purpose we performed a time course experiment with or without U0126 treatment of RD cells that were subsequently processed for FACS and immunoblotting analysis. As shown in Figure 2A, while c-Myc level (grey bars) was already significantly reduced at 3 hrs of U0126 treatment the percentage of cells in G0/G1 (black bars) was unchanged compared to untreated cells. Subsequently at 12 hrs, the percentage of cells in G0/G1 phase was highly increased by U0126 treatment, thus following of several hours the c-Myc down-regulation (Fig 2A compare 3 hrs with 12 hrs). This result demonstrated that, since in the U0126-treated cells the loss of c-Myc preceded their withdrawal from cell cycle, c-Myc down-regulation is not a consequence of the cessation of cell growth but rather it might cause growth arrest. In light of these results we hypothesised that c-Myc-dependent cell cycle proteins expression was altered too. In fact, it has been suggested that c-Myc-mediated cell transformation involves modulation of cell cycle protein expression [13,15,17]. Thus, we investigated whether cell cycle proteins were modulated in U0126-treated RD cells by immunoblotting experiments. Figure 2B shows that U0126 treatment induced a decrease in cyclin E2, which was stronger than that observed in cyclin E1, from 12 hrs up to 4 days, and a decrease in cyclin A and B accumulation which started at 1 day and persisted thereafter (2–4 days). Moreover, a reduction in CDK2, which forms complexes with cyclin E, A and B, started one day after treatment (Fig 2B). Of note, we have recently shown that U0126 induced a decrease in cyclin D1 and an increase in CKI, p21^WAF1 ^and p27 [27]. Lastly, the expression profile of the cyclins, CDK and CKI was in agreement with the hypo-phosphorylated/active form of pRb, which was detected as early as 12 hrs after treatment started (Fig 2B). These results point to the existence of a pathway in which an U0126-mediated lack of c-Myc activity affects cell cycle protein expression and mediates G0/G1 cell cycle arrest in RD cells.

### Blockade of functional c-Myc induces growth arrest

**Figure 3:**
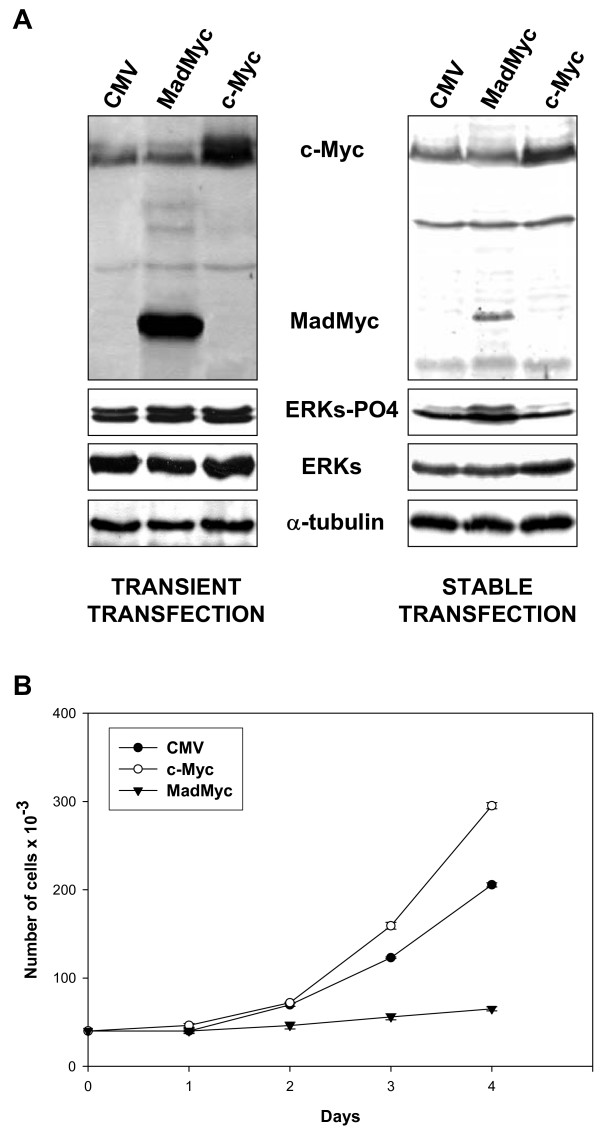
Cell cycle arrest by MadMyc chimera expression in RD cells. **A**. Cell lysates from polyclonal population of CMV, MadMyc chimera and c-Myc transiently and stably transfected RD cells were analysed by immunoblotting with c-Myc monoclonal antibody recognising both c-Myc and MadMyc chimera proteins. Same filters were re-probed with pospho-ERKs, ERKs and α-tubulin antibodies. **B**. Growth curve of polyclonal populations of RD cells stably transfected with MadMyc chimera (▼), c-Myc (○), and CMV (●). Polyclonal populations of stably transfected RD cells were plated and counted at indicated times. The data show the mean ± s.e.m. of triplicates of a representative experiment. Similar results were obtained in two experiments.

**Figure 4:**
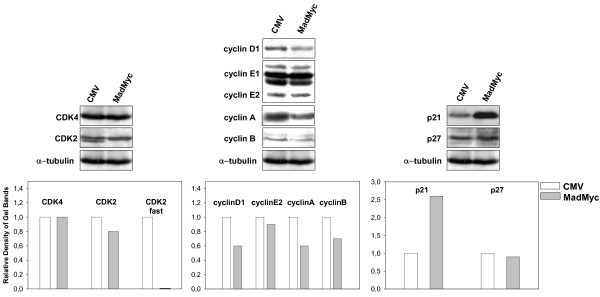
Effects of MadMyc chimera expression on CDK, cyclin and CKI proteins. Polyclonal populations of RD cells stably transfected with MadMyc chimera and CMV were plated and harvested after 4 days. Cell lysates were analysed by immunoblotting with specific antibodies for indicated proteins. α-tubulin expression shows equal loading. The histograms (lower panel) show the values of fold increase over the control, arbitrarly set at 1, obtained by densitometric analysis of the immunoblottings. Similar results were obtained in two experiments.

In order to verify whether in RD cells loss of c-Myc might cause growth arrest (Fig 1A) in the absence of MEK/ERK inhibition by U0126, we stably transfected RD cells with vector expressing MadMyc chimera, a strong antagonist of c-Myc activity [15]. RD cells stably transfected with c-Myc expressing vector and vector alone were also prepared. The efficiency of MadMyc chimera and c-Myc transfections was assessed by immunoblotting of transient and stably transfected RD cells with c-Myc antibody, which recognizes both c-Myc and MadMyc chimera (Fig 3A). Phospho-ERK immunoblotting revealed that there were more phospho-ERKs in MadMyc stably transfected cells than in either c-Myc- or CMV-transfected cells (Fig 3A), whereas no changes were detected in transiently transfected samples. The stably transfected polyclonal populations were also analysed for growth potential (Fig 3B). Proliferation of MadMyc-expressing cells was reduced after plating by 33.7% on day 2, and up to 68.4% by day 4, thereby indicating that MadMyc chimera expression blocked RD cell proliferation. By contrast, c-Myc over-expressing cells proliferated more than control cells (CMV) from day 3, attaining a 43.6% increase over the level of control cells by day 4. In MadMyc chimera stably transfected cells, expression of cyclin D1, A and B as well as the faster migrating form of CDK2 [21], which is present in CMV, were markedly reduced, whereas CDK4 expression was not (Fig 4). Moreover, increased p21^WAF1 ^expression occurred in MadMyc-expressing cells (Fig 4). These data demonstrate that c-Myc pathway disruption (MadMyc chimera expression) determines a molecular pattern resembling that induced by the MEK/ERK inhibitor (Fig 2). However, cyclin E1, E2 and p27 were not altered by MadMyc expression (Fig 4), suggesting that cyclin E down-regulation and p27 enhanced expression by U0126 might be due to ERK depletion [34] in RD cells. Taken together, these data demonstrate that c-Myc pathway disruption alone (MadMyc chimera expression) establishes a molecular pathway for growth arrest in RD cells.

### Anchorage-independent growth of RD cells is inhibited by U0126-mediated c-Myc down regulation and rescued by c-Myc over-expression

We have previously shown that RD cell growth inhibition can be induced by phorbol ester TPA and U0126 through different mechanisms mediated by ERK activation and inhibition respectively [27,28].

**Figure 5:**
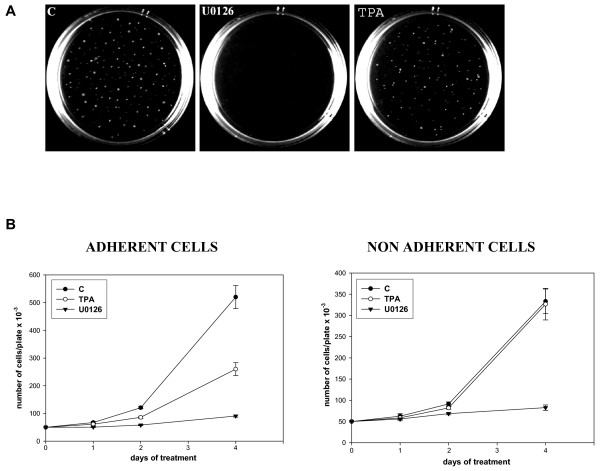
Effects of U0126 and TPA on anchorage independent growth of RD cells. **A**. RD cells left untreated (C) or treated with U0126 or 10^-7 ^M TPA were tested for growth in soft agar. Colonies were photographed after 14 days. **B**. Growth curves of adherent and non adherent RD cells left untreated (●) or treated with U0126 (▼) or TPA (○). RD cells were counted at indicated times. The data shown are the mean ± s.e.m. of triplicates of a representative experiment. Similar results were obtained in three experiments for A and two for B.

We therefore investigated whether the growth inhibitory function of U0126 and TPA was accompanied by a decreased anchorage independent growth, as determined by a colony-forming assay in soft agar. No colony formation was observed in U0126-treated cells after 2 weeks, whereas numerous, large colonies were present in both the untreated and TPA-treated RD cells (Fig 5A). These data demonstrate that U0126, though not TPA, inhibits anchorage-independent growth in RD cells.

**Figure 6:**
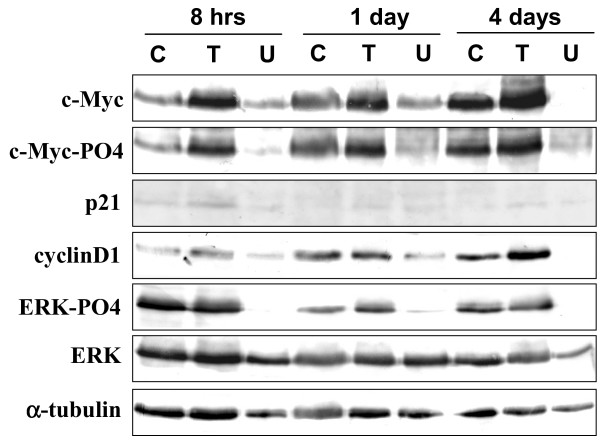
Effects of U0126 and TPA on the expression of c-Myc, p21^WAF1^, cyclin D1 and ERKs of RD cells grown in suspension culture. Cell lysates from cells left untreated (C) or treated with U0126 (U) or TPA (T) for indicated times were analysed by immuoblotting with specific antibodies for indicated proteins. α-tubulin expression shows the loading of samples. Similar results were obtained in two different experiments.

In order to explain the different effects of U0126 and TPA in regulating anchorage-independent growth, we investigated whether cells growing without substrate attachment were still responsive to growth arrest signals induced by U0126 and TPA. For this purpose we performed an experiment in which RD cells were grown either in suspension (see Methods) or in adherent cultures, in the presence or absence of U0126 or TPA. Growth was monitored at 1, 2 and 4 days. U0126 in both suspension and adherent cultures inhibited growth (Fig 5B), whereas TPA did not induce growth arrest in suspension, as instead occurred in adherent cultures (Fig 5B). These results demonstrated that the growth potential of RD cells can be inhibited by both TPA and U0126, whereas anchorage-independent growth is abolished by U0126 only. We then investigated whether the different effects of U0126 and TPA on growth without a substrate attachment can be correlated with the modulation of c-Myc phosphorylation and expression levels as observed in U0126-treated cells (Fig 1). We found that c-Myc expression and phosphorylation were enhanced after TPA treatment and were instead down-regulated by U0126 (Fig 6 and see additional file 2). We also analysed the levels of p21^WAF1 ^and cyclin D1, previously shown to be up-regulated in response to TPA-mediated growth arrest signal [27]. While U0126 down-regulated cyclin D1, TPA, according to its inability to arrest growth in suspension culture, failed to increase the levels of p21^WAF1 ^but still induced cyclin D1 increased expression. It is noteworthy that, in this culture condition, TPA only slightly stimulated ERK phosphorylation whereas U0126 still inhibited phospho-ERK levels. The results of growth with or without substrate attachment (Fig 5) and of the biochemical analysis (Fig 6), demonstrate that the mere growth potential inhibition in adherence condition is not a requisite for the reversal of anchorage independent growth.

**Figure 7:**
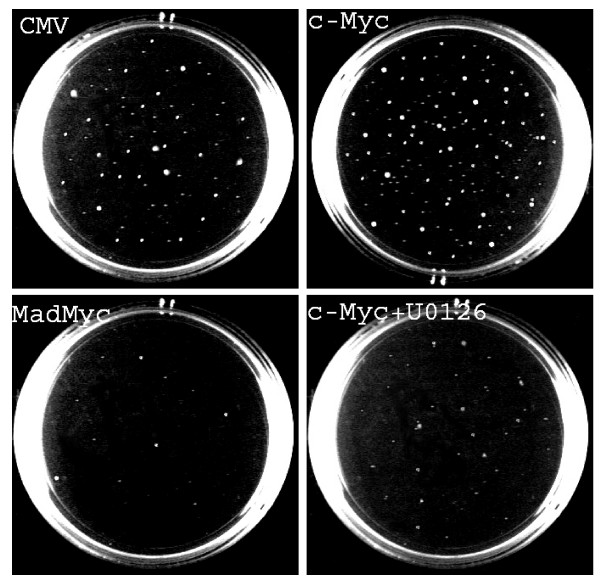
Effects of c-myc on anchorage independent growth of RD cells. Polyclonal populations of RD cells stably transfected with MadMyc chimera, c-Myc and CMV vectors were tested for growth in soft agar. c-Myc polyclonal populations were left untreated or treated with U0126. Colonies were photographed after 14 days. Similar results were obtained in two experiments.

The results of Figure 5 and 6 suggested to assess whether c-Myc pathways played by itself a role in the reversal of anchorage-independent growth in the absence of MEK/ERK inhibition. To this purpose c-Myc- and MadMyc chimera-transfected clones were grown in soft agar. The results of the soft agar assay of CMV-, c-Myc- and MadMyc chimera-stably transfected cells demonstrated that MadMyc chimera expression abolished, whereas c-Myc expression enhanced, colony formation, when compared with CMV-transfected cells (Fig 7). Furthermore, c-Myc overexpression sensibly rescued the anchorage-independent growth in U0126-treated cells (Fig 7). The results of MadMyc chimera indicate that the disruption of the c-Myc can be responsible of the reversion of transformation potential in RD cells.

### Molecular and morphological myogenic-like phenotype is induced by MadMyc chimera and is attenuated by forced c-Myc expression

**Figure 8:**
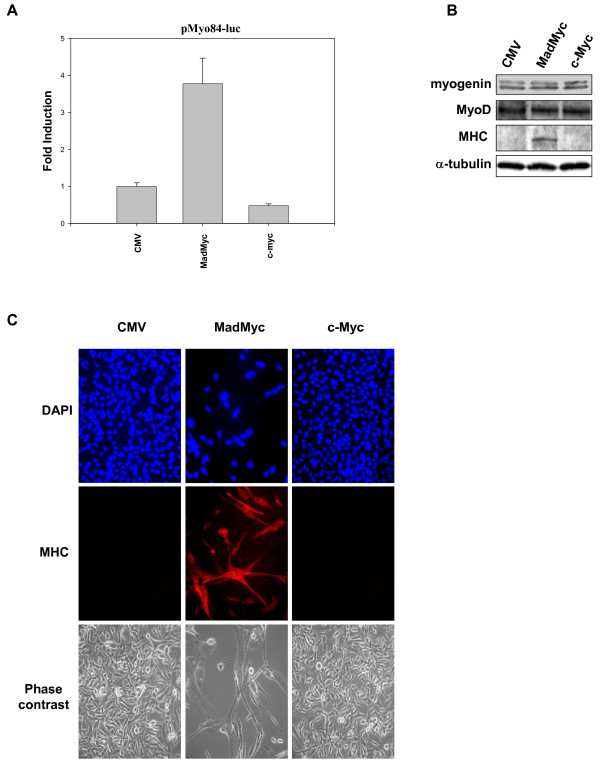
Activation of myogenic program in MadMyc chimera transiently transfected RD cells. **A**. Luciferase assay of lysate from RD cells co-transfected with the empty vector (CMV) or MadMyc- or c-Myc-expressing vectors and the plasmid carrying myogenin promoter (pMyo84-luc). The histogram shows the fold induction of myogenin promoter (pMyo84-luc) of MadMyc- or c-Myc- versus CMV-transfected RD cells arbitrarily set at 1. Data show mean values ± s.e.m. of triplicates of two experiments. **B**. Immunoblotting of parallel culture as in (A) with antibodies for indicated proteins. **C**. Immunofluorescence with antibody directed to sarcomeric myosin (MHC) of RD cells stably transfected with MadMyc chimera, c-Myc and CMV after 4 days of plating (middle panels). Cell nuclei were stained with DAPI (upper panels). Photomicrographs by contrast phase (lower panels) of other fields. Similar results were obtained in two experiments.

Since c-Myc over-expression efficiently inhibits myogenesis [35], we investigated whether the functional inactivation of c-Myc rescued the myogenic program. For this purpose, RD cells were transiently co-transfected with MadMyc chimera- or c-Myc-expressing vector together with a reporter plasmid (pMyo84-luc) containing the MEF and E-box binding sites of human myogenin promoter [36]. We observed a four-fold increase in the myogenin promoter transactivation as a result of MadMyc chimera expression (Fig 8A). By contrast, c-Myc over-expression (c-Myc) led to a significant (two-fold) reduction in basal myogenin promoter activity (CMV). Moreover, no changes in myogenin or MyoD expression levels occurred in either MadMyc chimera- or c-Myc-transfected cells (Fig 8B), suggesting that MadMyc chimera expression led to the rescue of myogenic transcription factor transactivating functions. Expression of myogenic-specific markers, such as sarcomeric myosin heavy chain (MHC), occurred as a result of the restored function of myogenic transcription factors (Fig 8B).

**Figure 9:**
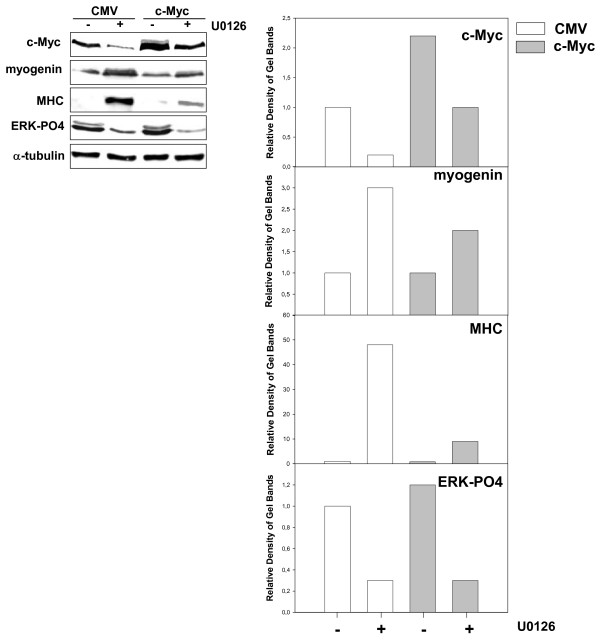
c-Myc overexpression attenuates U0126 differentiative effects. Immunoblotting with specific antibodies for indicated proteins of cell lysates from c-Myc polyclonal population of RD cells left untreated or treated with U0126. α-tubulin expression shows the loading of samples. The histograms (right panel) show the values of fold increases over the control, arbitrarly set at 1, obtained by densitometric analysis of the immunoblottings. Similar results were obtained in two experiments.

Furthermore, MadMyc chimera stably-expressing cells predominantly displayed an elongated myotube-like cell morphology, as shown in the immunofluorescence experiment with MHC antibody (Fig 8C). Lastly, in order to ascertain whether the over-expression of c-Myc overcame the differentiative effect of U0126, RD cells transiently transfected with c-Myc or empty (CMV) vectors were treated with U0126, or were left untreated, for 4 days, and were analysed for c-Myc, phospho-ERK, myogenin and sarcomeric myosin expression. The results demonstrated that U0126 inhibited phospho-ERKs in both CMV- and c-Myc transfected cells, markedly down- regulated c-Myc, and increased myogenin and myosin expression in CMV-transfected cells. By contrast, c-Myc forced expression attenuated U0126-mediated c-Myc down-regulation, myogenin and myosin increased expression (Fig 9). This result suggested that the U0126-mediated effects on the myogenic program were counteracted by the high c-Myc level.

Taken together, these results demonstrate that the mere inhibition of c-Myc can rescue the myogenic program in RD cells by myogenic transcription factor activation, MHC expression and myogenic-like phenotype acquisition.

### U0126 down-regulates c-Myc and counteracts the oncophenotype of non-muscle-derived tumor cell lines

**Figure 10:**
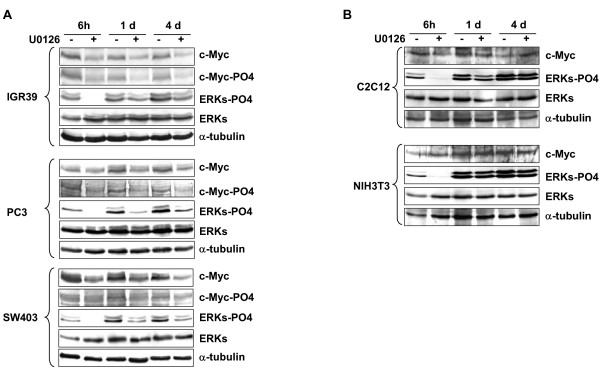
Down-regulation of c-Myc by U0126 in non-muscle tumor cell lines. **A**. Total lysates from the indicated non-muscle tumor cell lines untreated (-) or treated (+) with 10 μM U0126 for indicated times were analysed by immunoblotting with anti-c-Myc, anti-pospho-c-Myc, anti-ERKs and anti pospho-ERKs. **B**. Total lysates from untransformed C2C12 and NIH3T3 were analysed by immunoblotting with anti-c-Myc, anti-ERKs and anti pospho-ERKs. α-tubulin expression shows the loading of samples. Similar results were obtained in two experiments.

**Figure 11:**
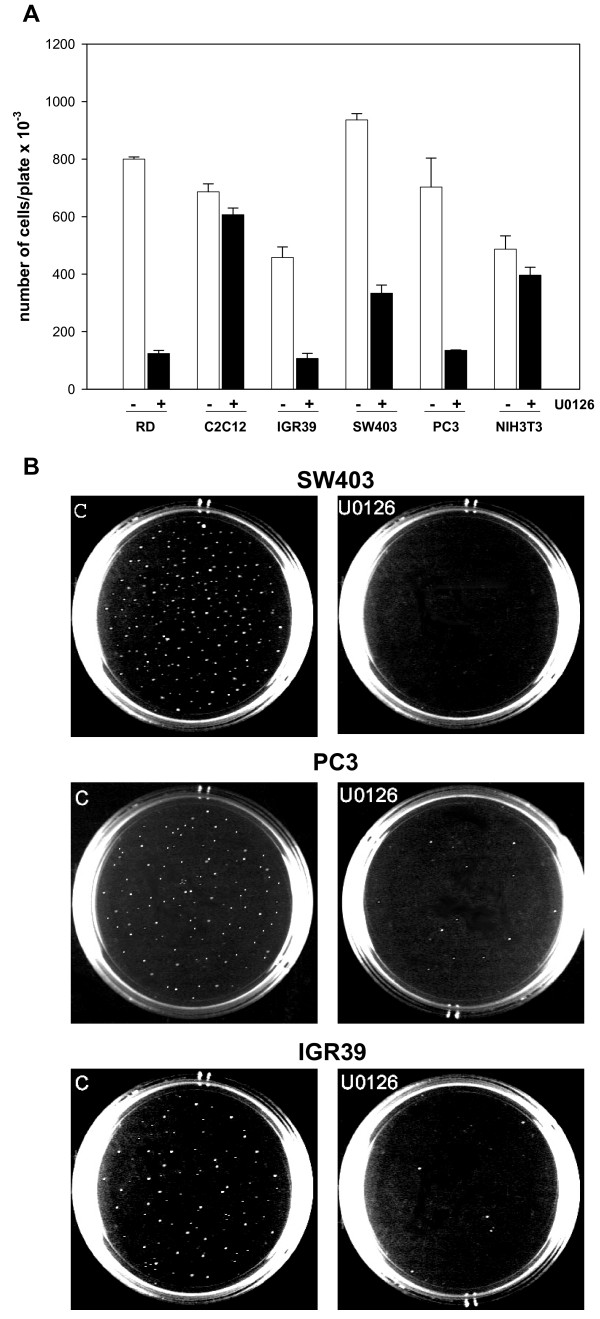
Effects of U0126 on non-muscle tumor cell lines proliferation and anchorage-independent growth. **A**. The histogram shows the number of cells from tumor cell lines and, as control, from untransformed C2C12 and NIH3T3, cultured for 4 days with (+) or without (-) U0126. The data shown are the mean ± s.e.m. of triplicates of a representative experiment. **B**. Tumor cells left untreated (C) or treated with U0126 were tested for growth in soft agar. Colonies were photographed after 14 days. Similar results were obtained in two experiments.

To investigate whether the anti-growth and anti-oncogenic effects of MEK/ERK inhibition are peculiarity of soft tissue-derived tumor cell lines, such as RD, we used IGR39 melanoma-, SW403 colon adenocarcinoma-, PC3 prostate-derived human tumor cell lines, C2C12 and NI3T3 as control untransformed muscle and non-muscle cell lines. We first investigated, in time course experiments either with or without U0126, the effects of MEK/ERK inhibition on the c-Myc phosphorylation level and expression. As shown in Figure 10A (see additional file 3), U0126 efficiently inhibited ERK phosphorylation in all the tumor cell lines tested and induced a decrease in c-Myc expression as well as in its phosphorylation throughout the treatment period (6 hrs-4 days). In the normal cell lines, such as C2C12 and NIH3T3, phospho-ERK was markedly inhibited by U0126 at early treatments (6 hrs), but recovered at longer treatments (1–4 days). U0126 treatment did not alter c-Myc expression in either C2C12 or NIH3T3 (Fig 10B). The analysis of growth potential (Fig 11A) demonstrated that U0126 treatment reduced, as in RD cells, the number of cells by 71% in IGR39, 65% in SW403 and 81% in PC3 cells. Normal untransformed cell lines were less sensitive to the growth inhibiting effects of U0126, with the number of cells dropping by 12% in C2C12 and 18% in NIH3T3. These results indicated that in normal untransformed cell lines U0126 inhibited growth slightly, while failed to induce long-lasting phospho-ERK inhibition.

Moreover, the colony-forming assay in soft agar showed that the colony formation of the IGR39, SW403 and PC3 tumor cell lines was abolished by U0126, whereas numerous, large colonies were present in the untreated cells (Fig 11B).

These data show that cell transformation of different tumor-derived cell lines is halted by inhibition of MEK/ERK pathway followed by c-Myc down-regulation.

## Discussion

The pharmacological inhibitors of Ras/MEK/ERK signalling are arousing considerable interest on account of their potential therapeutic uses [22,37]. In this paper, we addressed the issue of whether MEK/ERK inhibition, by targeting c-Myc, prevents the transformed phenotype expression in RD cells as well as in a number of tumor cell lines that express a mutated version of ras and over-express c-Myc. The efficient growth inhibition induced by the MEK inhibitor U0126 in RD, colon carcinoma, prostate and melanoma cell lines clearly demonstrates that the MEK/ERK pathway is a pre-requisite for the aberrant growth of these cells. Indeed, U0126 permanently inhibits phospho-ERKs in all tumor cell lines used. It is noteworthy that both c-Myc phosphorylation and c-Myc expression itself decreased in RD cells as well as in all the non muscle tumor cell lines examined following MEK/ERK inhibition. Conversely, in muscle and non-muscle untransformed cell lines, U0126, while transiently inhibiting phospho-ERKs, only slightly inhibits growth and does not down-regulate c-Myc. This result is consistent with no major effects of MEK/ERK inhibition on proliferation status of muscle and non-muscle untransformed cell lines. All together these data are in line with the notion that c-Myc is a downstream target of MEK/ERK pathway and suggest that aberrant growth of different tumor cell lines can be halted by targeting c-Myc following MEK/ERK inhibition. Although c-Myc has previously been reported to be a downstream target of MEKs/ERKs [7] the correlation between ERK-mediated c-Myc stability and aberrant growth, though inferable from recent studies in the literature [37,38], has so far received little attention.

Besides inducing growth arrest, U0126 also abolished, in the cell lines used here, anchorage-independent growth, as demonstrated by the lack of clones in the soft agar assay. In addition, in RD cells the comparison of growth in soft agar in the presence of U0126 or TPA demonstrates that while TPA only reduces the growth potential of RD cells, U0126 is also able to abolish anchorage-independent growth. The failure of TPA to abolish anchorage-independent growth can be explained by its inability to induce p21^WAF1 ^and its positive effects on c-Myc and cyclin D1 expression in non-adherent RD cultures. Conversely, the U0126-mediated arrest of growth in non-adherent cultures can be due to the drastic c-Myc down-regulation and cyclin D1, known to be involved in cell transformation [12,16,39]. In addition, the experiment in suspension cultures suggest that MEK/ERK inhibitor, U0126, may have cytostatic effects [40]. These results demonstrate that the mere inhibition of growth potential is not sufficient to prevent the transformed phenotype expression.

Recent studies in the literature report, on the one hand, that MAPKs and c-Myc cooperate in promoting invasive growth [41] and, on the other, that targeted disruption of c-Myc suppresses cell transformation and tumor formation [42]. The Ras-MAPK pathways are, however, currently receiving attention owing to the therapy potential they offer [37], while a number of papers reporting that c-Myc inactivation results in tumor inhibition and regression [11,12,18]. Our data attempt to demonstrate a possible link between these two major targets in a cascade in which MEK/ERK kinases lie upstream of the oncogenic molecule c-Myc which, in turn, induces neoplastic transformation. In fact, we here show that ERKs and particularly ERK2, are upstream kinases of c-Myc in RD cells as demonstrated by siRNA results. These results are in line with data reported by others that c-Myc stability and accumulation is regulated by ERK-mediated phosphorylation of ser62 [43]. Moreover, it is evident the relationship between MEK/ERK inhibition, c-Myc down regulation and blockade of cell transformation in the cell lines here used. This functional correlation is highly relevant in the field of possible new therapeutic approaches for some human tumor, including rhabdomyosarcoma.

In an attempt to determine the specific role of c-Myc in sustaining aberrant growth as well as cell transformation and inhibition of differentiation, we used RD cells on account of their ability to undergo growth arrest and myogenic differentiation upon MEK/ERK inhibition [27,28]. Our data show that MEK/ERK inhibition down-regulates cyclin E2, A and B and CDK2, all of which are known to be transcriptional targets of c-Myc [13,15,44]. It can, consequently, be hypothesized that the disruption of the c-Myc network by ERK depletion is responsible for the failed expression of the relevant cell-cycle proteins.

Hypothesising that c-Myc expression alone sustains the program for deregulated growth as well as transformation and inhibition of differentiation, we stably over-expressed MadMyc chimera in RD cells to specifically block c-Myc activity [15]. We found that growth of MadMyc-over-expressing RD cells is arrested, as demonstrated by p21^WAF1 ^enhanced expression and cyclin D1, A and B and CDK2 down-regulation, as also observed in U0126-treated cells. Furthermore, myogenic differentiation is induced in MadMyc-expressing RD cells, as shown in this study by the restored transcriptional function of myogenic transcription factors and MHC expression. It is noteworthy that induction of myogenic differentiation in MadMyc chimera expressing cells does not imply a myogenin or MyoD increased expression level neither down-regulation of pospho-ERKs which are instead enhanced. This is in agreement with the role of ERKs in fusion and late differentiation processes during myogenic differentiation [45]. Importantly, MadMyc-stably-expressing cells do not exhibit anchorage-independent growth, which is instead enhanced in c-Myc-over-expressing cells. On the other hand, forced expression of c-Myc attenuated the U0126-mediated anchorage-independent growth inhibition and differentiative effects in RD cells. These experiments demonstrate that c-Myc over-expression rescues oncogenic phenotype repressed by MEK inhibitor U0126. Worthy of note is also the fact that the role of mutated Ras in aberrant growth of RD cells is compromised by the selective disruption of c-Myc in MadMyc-expressing cells demonstrating that c-Myc is indispensable to the maintaining of Ras/MEK/ERK-mediated oncogenic phenotype.

## Conclusion

Our data provide evidence that the cooperation between MEK/ERK and c-Myc pathways play a major role in the expression of transformed phenotype in muscle and non muscle-derived transformed cell lines. Importantly, our results show for the first time that the disruption of c-Myc pathway either directly or indirectly drammatically impairs the expression of transformed phenotype inducing myogenic differentiation in RD cells. In conclusion these data strongly suggest that the targeting of c-Myc by means of the MEK/ERK inhibitor can be tested as a promising strategy in anti-cancer therapy.

## Methods

### Cell cultures and treatments

The embryonal Rhabdomyosarcoma (RD), the prostate carcinoma PC3 (ATCC, Rockville MD), the melanoma IGR39 and colon adenocarcinoma SW403 (DSMZ, Braunschweig, Germany) human cancer cell lines were cultured in Dulbecco modified Eagle medium (DMEM), supplemented with glutamine, gentamycin (GIBCO-BRL Gaithersburg, MD) and 10% (RD, PC3 and SW403) or 15% (IGR39) heat-inactivated foetal bovine serum (Hyclone, Logan UT). C2C12 and NIH3T3 (ATCC) were grown in DMEM supplemented with glutamine, gentamycin and 10% heat-inactivated foetal bovine serum. One day after plating, cells were treated with 10 μM U0126 kinase inhibitors (Promega, Madison, WI) or 10^-7 ^M TPA (Sigma, St Louis, MO) for the times shown in the figures.

### Immunoprecipitation

Cells were harvested in phosphate buffered saline, sedimented and lysed in 10 mM Tris pH 7, 50 mM NaCl, 1% NP40, 1 mM ZnCl2, additioned with protease and phosphatase inhibitors. Protein extracts were clarified by centrifugation. Supernatant, normalized as equal amounts of proteins, were incubated with Max antibody (H-2) (Santa Cruz Biotechnology, Santa Cruz CA) at 4°C for 3 hrs. 30 μl of protein-G Plus (Santa Cruz Biotechnology) were added to collect immunocomplexes. Protein G-bound immunocomplexes were washed 6 times with extraction buffer and processed for SDS-PAGE and immunoblotting.

### Immunoblot analysis

Cells were lysed in 2% SDS containing phosphatase and protease inhibitors sonicated for 30 sec. Proteins of whole cell lysates were assessed using the Lowry method [46], and equal amounts were separated on SDS-PAGE. The proteins were transferred to a nitrocellulose membrane (Schleicher & Schuell, BioScience GmbH, Germany) by electroblotting. Immunoblottings were performed with the following antibodies: anti-c-Myc polyclonal (N-262) or monoclonal (9E10), anti-phospho c-Myc (Thr 58/Ser 62-R), anti-Max (H-2), anti-phospho ERK1/2 (E-4), anti-ERK2 (C-14 positive also for ERK1), anti-p21^WAF1 ^(C-19), anti-p27 (F-8), anti-Cyclin-E (HE12), -A (H-432), -D1 (M 20) and -B (H-20), CDK2 (M2) and 4 (H-22), -pRb (C-15), anti-myogenin (F-D5), a-tubulin (B-7), MyoD (C-20) (all from Santa Cruz Biotechnology) and anti-MHC (MF20, gift from Fichman D). Peroxidase-conjugate anti-mouse or anti-rabbit IgG (Amersham-Pharmacia Biotech, UK or Santa Cruz) were used for enhanced chemiluminescence (ECL) detection.

### Plasmids and transfection

One day after plating, RD cells were transfected with plasmids using Lipofectamine Plus reagent (Invitrogen, Italy) according to the manufacturer's instructions. For the luciferase assay, the CMV or the c-Myc (kindly provided by Dr. L.G. Larsson) or MadMyc chimera plasmid (kindly provided by Dr. R. Bernards) were co-tranfected in RD cells together with pMyo84-luc (kindly provided by B.M. Scicchitano described in [36]). Total lysates were processed for luciferase activity according to the manufacturer's instructions (Promega Italia).

RD stably transfected cells were obtained transfecting cells with a plasmid encoding c-Myc, MadMyc chimera or empty vector CMV, all carrying G418-neomycin resistance. Polyclonal populations of CMV, c-Myc and MadMyc chimera expressing cells were selected using 0.4 mg/ml of G418-neomycin (Sigma) for three weeks. RNA interference experiments were performed with siRNA for ERK1 and ERK2 (Sancta Cruz Biotechnology) using Lipofectamine 2000 reagent (Invitrogen, Italy), according to the manufacturer's instructions. Briefly, cells were plated at 40–50% confluence and transfected after 24 hr with 100 nM siRNA, which we ascertained was sufficient to detect maximum fluorescence using fluorescein-conjugated control siRNA.

### Immunofluorescence

Cells were fixed in 4% paraformaldehyde and washed; non-specific binding sites were blocked with 3% BSA in PBS for 20 min at room temperature. Cells were then incubated for 1 hr at RT with a 1:100 dilution of the anti-MHC (MF20), specific mouse monoclonal antibody. After rinsing with PBS, the cells were incubated with anti-mouse IgG-Cy3 and DAPI (Zymed, Invitrogen, Italia).

### Suspension cell cultures and colony-forming assays in semisolid agar

RD cells were initiated as adherent cultures, detached and seeded in 50 ml Falcon tube at 5 × 10^4 ^cells/ml in a total volume of 12 ml of same medium as adherent cultures and after 1 day additioned with TPA or U0126. All tubes were placed in an orbital shaker (~120 rpm) in a 37°C humidified incubator with 5% CO_2_.

Colony-forming assays were based on standard methods. Briefly, 2 × 10^4 ^cells were resuspended in 4 ml of 0.33% special Noble agar (Difco, Detroit, MI) and plated (6 cm plate) in growth medium-containing 0.5% soft agar. Colonies were photographed 14 days after plating.

### Cell proliferation assay and FACS analysis

Cells from adherent and suspension culture were counted using hemocytometer, and tested for exclusion of trypan blue. Data are expressed as average of triplicate + standard error.

For FACS analysis cells were harvested by trypsin-EDTA and washed; pellets were resuspended in 0,3 ml 50% FCS in PBS, additioned with 0,9 ml 70% ethanol and left O/N in the dark at 4°C before FACS analysis (Coulter Epics XL Flow Cytometer, Beckman Coulter CA, USA).

## Abbreviations

RD, rhabdomyosarcoma cell line; MEK, Mitogen-activated protein Extracellular Kinase; ERK, Extracellular signal-Regulated protein Kinase.

## Authors' contributions

FM contributed to the acquisition of most of the data presented. CC contributed to the acquisition of data regarding non muscle cell lines. BMZ, conceived the study and wrote the manuscript.

## Supplementary Material

Additional File 1 U0126-mediated phospho-ERK inhibition during culture times. Immunoblotting of cell lysates from untreated (-) and treated (+) cells with U0126 using antibodies recognizing phospho-ERKs and total ERKs. Click here for file

Additional File 2 Quantitative analysis of immunoblotting of Figure 6. The values of fold increases over the control, arbitrarly set at 1, are obtained by densitometric analysis. Click here for file

Additional File 3 Quantitative analysis of immunoblotting of Figure 10. The values of fold increases over the control, arbitrarly set at 1, are obtained by densitometric analysis. Click here for file
