# Supplementary figures and images for: Down-regulation of c-Myc following MEK/ERK inhibition halts the expression of malignant phenotype in rhabdomyosarcoma and in non muscle-derived human tumors
Source: Mol Cancer. 2006 Aug 9;5:31. doi: 10.1186/1476-4598-5-31 (PMC1560159; doi:10.1186/1476-4598-5-31)

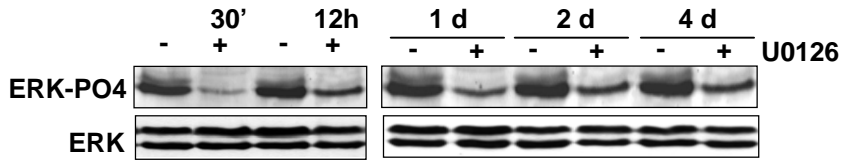

Supplement: Additional File 1 — U0126-mediated phospho-ERK inhibition during culture times. Immunoblotting of cell lysates from untreated (-) and treated (+) cells with U0126 using antibodies recognizing phospho-ERKs and total ERKs. [file 1476-4598-5-31-S1.pdf]
