# Supplementary material for: Down-regulation of c-Myc following MEK/ERK inhibition halts the expression of malignant phenotype in rhabdomyosarcoma and in non muscle-derived human tumors
Source: Mol Cancer. 2006 Aug 9;5:31. doi: 10.1186/1476-4598-5-31 (PMC1560159; doi:10.1186/1476-4598-5-31)
Supplement: Additional File 2 — Quantitative analysis of immunoblotting of Figure 6. The values of fold increases over the control, arbitrarly set at 1, are obtained by densitometric analysis. [file 1476-4598-5-31-S2.pdf]

	8 hrs			1 day			4 days		
	C	T	U	C	T	U	C	T	U
c-Myc	1	2.7	1	1	1.2	0.6	1	1.7	0
c-Myc-PO4	1	2.6	0.3	1	1.1	0.9	1	1.2	0.3
p21	1	1.2	1	1	3	2.6	1	1.2	0.6
cyclinD1	1	2.5	1.1	1	0.9	0.3	1	2.3	0
ERK-PO4	1	1	0	1	2	0.1	1	1.2	0
ERK	1	1	1	1	1	1.2	1	1	0.8
