# Supplementary material for: Down-regulation of c-Myc following MEK/ERK inhibition halts the expression of malignant phenotype in rhabdomyosarcoma and in non muscle-derived human tumors
Source: Mol Cancer. 2006 Aug 9;5:31. doi: 10.1186/1476-4598-5-31 (PMC1560159; doi:10.1186/1476-4598-5-31)
Supplement: Additional File 3 — Quantitative analysis of immunoblotting of Figure 10. The values of fold increases over the control, arbitrarly set at 1, are obtained by densitometric analysis. [file 1476-4598-5-31-S3.pdf]

| | IGR39 | | PC3 | | SW403 | |
|---|---|---|---|---|---|---|
| | c-Myc | c-Myc-PO4 | c-Myc | c-Myc-PO4 | c-Myc | c-Myc-PO4 |
| **C 6h** | 1 | 1 | 1 | 1 | 1 | 1 |
| **U0126 6h** | 0.5 | 0.2 | 0.7 | 0.5 | 0.5 | 0.6 |
| **C 1d** | 1 | 1 | 1 | 1 | 1 | 1 |
| **U0126 1d** | 0.5 | 0.5 | 0.6 | 0.5 | 0.5 | 0.6 |
| **C 4d** | 1 | 1 | 1 | 1 | 1 | 1 |
| **U0126 4d** | 0.2 | 0.5 | 0.5 | 0.3 | 0.3 | 0.6 |
